# Supplementary material for: SyNDI: synchronous network data integration framework
Source: BMC Bioinformatics. 2018 Nov 6;19:403. doi: 10.1186/s12859-018-2426-5 (PMC6219086; doi:10.1186/s12859-018-2426-5)
Supplement: Supplementary file 5 — Files of the example presented in Additional file 4. This additional file is a zip package that contains all files used in Additional file 4. Detailed descriptions of each of these files are presented in a README file included in this zip package. (GZ 30411 kb) [file 12859_2018_2426_MOESM5_ESM.gz › SyNDITools/Meme2Fimo/meme_4.9.0_4.tar.gz!/meme_4.9.0/doc/motiph.xml]

motiph


> # motiph
>
>   
> **Usage:**
> > `motiph [options] <alignment file> <tree file> <motif file>`
>
> **Description:**
>
> > Scan a DNA multiple sequence alignment for occurrences of given motifs,
> > taking into account the phylogenetic tree relating the sequences.
> > **Note:** any sequences containing nothing but gaps are removed from the
> > alignment.
> >
> > The algorithm is as follows:
> >
> > ```
> >       A = a multiple alignment
> >       T = a phylogenetic tree
> >       M = a motif frequency matrix
> >       B = the background frequencies of the four bases in A
> >       U = a background evolutionary model with equilibrium frequencies B
> >
> >       // Build evolutionary models.
> >       for j in positions of M {
> >         E[j] = an evolutionary model with equilibrium frequencies 
> >           from the jth position of M
> >       }
> >
> >       // Build matrix of all possible scores
> >       for i in all possible alignment columns {
> >         for j in positions of M {
> >           foreground = site_likelihood(E[j], A[:][i], T)
> >           background = site_likelihood(U, A[:][i] T)
> >           score_matrix[i][j] = log_2(foreground / background)
> >         }    
> >       }
> >       // Calculate p-values of all possible scores for motif sized windows
> >       // windows in the alignment.
> >       pvalues = get_pv_lookup(score_matrix, B)
> >
> >       // Calculate score for each motif sized window in the alignment.
> >       for i in columns of A {
> >         score = 0
> >         for j in positions of M {
> >           index = calculate the index of A[:][i + j] in the array
> >             of all possible alignment columns
> >           score = score + score_matrix[index][j]
> >         }    
> >         print pvalues[score]
> >       }
> > ```
> >
> > The core of the algorithm is a routine (`site_likelihood`)
> > for scoring a particular column of the multiple alignment using a
> > given evolutionary model and a given phylogenetic tree. The alignment
> > site provides the observed nucleotides at the base of the tree, and we
> > sum over the unobserved nucleotides in the rest of the tree,
> > conditioning on the equilibrium distribution from the evolutionary
> > model at the root of the tree (Felsenstein Pruning Algorithm). The
> > tree must be a maximum likelihood tree, of the kind generated by DNAml
> > from Phylip or by FastDNAml. Branch lengths in the tree are converted
> > to conditional probabilities using the specified evolutionary
> > model.
>
> **Input:**
>
> - `<alignment file>` is a DNA multiple alignment in
>   ClustalW format.
>   Alternatively, if the `--list` option is used,
>   this file may contain a list of alignment files.
>   In this case each of the alignment files will be scanned for
>   each of the motifs.
> - `<tree file>` is a phylogenetic tree in
>   Phylip Newick
>   format. This tree may contain
>   additional species not represented in the alignment.
> - `<motif file>` is a list of motifs, in
>   MEME format.
>
> **Output:**
> > The output is in
> > GFF
> > format with the forward and reverse scores listed as separate features.
> > The scores are the *p*-values for the observed alignment columns in the motif sized windows.
>
> **Options:**
>
>
>
>
>
>
>
>
>
>
>
>
>
>
>
>
>
>
>
>
>
>
>
>
>
> **Warning messages:** None
> **Bugs:**
>
> - F84 and Tamura-Nei models fail trying to take the log of a negative number.
> - Allow local realignment, a la Monkey.
> - The `--motif` option should allow multiple motifs
>   to be selected from the motif file.
> - Print motif consensus as part of feature properties.
> - Allow reading of a column frequency file; this requires
>   writing a stand-alone to create such a file and establishing
>   a format such as:
>
>   ```
>            # species_list_1
>            AAA f_AAA
>            ...
>            TTT f_TTT
>            # species_list_2
>            AAAAA f_AAAAA
>            ...
>            TTTTT f_TTTTT
>            ...
>   ```
